# *Anaplasma capra*: a new emerging tick-borne zoonotic pathogen

**DOI:** 10.1007/s11259-024-10337-9

**Published:** 2024-03-01

**Authors:** Kursat Altay, Ufuk Erol, Omer Faruk Sahin

**Affiliations:** Department of Parasitology, Faculty of Veterinary Medicine, Sivas Cumhuriyet University, Sivas, 58140 Türkiye

**Keywords:** *Anaplasma capra*, History, Hosts, Prevalence, Pathogenicity, Genotypes

## Abstract

The genus *Anaplasma* includes *A. marginale, A. centrale, A. bovis, A. ovis, A. platys*, and *A. phagocytophilum* transmitted by ticks, some of which are zoonotic and cause anaplasmosis in humans and animals. In 2012, a new species was discovered in goats in China. In 2015, the same agent was detected in humans in China, and it was provisionally named *Anaplasma capra*, referring to 2012. The studies conducted to date have revealed the existence of *A. capra* in humans, domestic animals, wild animals, and ticks from three different continents (Asia, Europe, and Africa). Phylogenetic analyses based on *gltA* and *groEL* sequences show that *A. capra* clearly includes two different genotypes (*A. capra* genotype-1 and *A. capra* genotype-2). Although *A. capra* human isolates are in the genotype-2 group, goat, sheep, and cattle isolates are in both groups, making it difficult to establish a host genotype-relationship. According to current data, it can be thought that human isolates are genotype-2 and while only genotype-1 is found in Europe, both genotypes are found in Asia. *Anaplasma capra* causes clinical disease in humans, but the situation is not yet sufficient to understand the zoonotic importance and pathogenicity in animals. In the present review, the history, hosts (vertebrates and ticks), molecular prevalence, pathogenic properties, and genetic diversity of *A. capra* were evaluated from a broad perspective.

## Introduction (the great progress in a short time in history of *Anaplasma capra*)

*Anaplasma* (family Anaplasmataceae, order Rickettsiales) species are obligate intracellular alphaproteobacteria that multiply within membrane-bound vacuoles and can cause disease in humans and a wide range of domestic animals (Dumler et al. 2001; Rar et al. 2021). The first clinical infections in animals were described in 1910 (Theiler 1910). Today, it still has effects on human and animal health at the global level (Rar et al. 2021). According to the classification based on *16S rRNA* and *groEL* genes, there are six *Anaplasma* species (*A. bovis, A. ovis, A. marginale, A. centrale, A. phagocytophilum*, and *A. platys*) in humans and animals (Dumler et al. 2001).

Developments in molecular genetics and the increased use of these techniques in the identification of pathogens have undoubtedly contributed greatly to the elucidation of the etiologies of diseases. With these techniques, different strains and genotypes of many pathogens have been revealed, progress has been made in their classification, and they have enabled the discovery of previously unidentified species (Dumler et al. 2001). In the study conducted in Central and Southern China in 2012, the isolate obtained from goats, which was different from other *Anaplasma* species according to its *16S rRNA* gene sequence, was recorded in the GenBank as Uncultured *Anaplasma* sp. (Liu et al. 2012). In 2015, in China, a novel *Anaplasma* species different from all known *Anaplasma* species was identified in 28 of 477 (6%) humans with tick bite history. It has been shown that the *16S rRNA* gene full sequence (1,499 bp) of this isolate, which is an important marker in genotyping, has 27–73 nucleotide differences with other *Anaplasma* species (Li et al. 2015). The species revealed in this study was named “*Anaplasma capra*” because goats were the host from which the agent was first isolated. In the study, *A. capra* was also detected in a tick species (*Ixodes persulcatus*) for the first time (Li et al. 2015). There is an important point to point out here; it is thought that *A. capra* was circulating in domestic and wild animals before 2012 when it was first reported. Phylogenetic analysis based on *16S rRNA* has shown that the species (*A. centrale* Aomori strain, AF283007, Inokuma et al. 2001) identified in cattle in Japan in 2001 is *A. capra* (Khumalo et al. 2018). Likewise, BLAST analyses of *A. centrale* (AB211164) detected in deer in Japan in 2005 (Kawahara et al. 2006) and *Anaplasma* sp. (AB509223) detected in serow in Japan in 2009 (Sato et al. 2009) revealed that they were *A. capra*. On the other hand, *A. capra* was detected in 2020 in blood samples taken from goats in 2011 (Zhang et al. 2020).

**Fig. 1:**
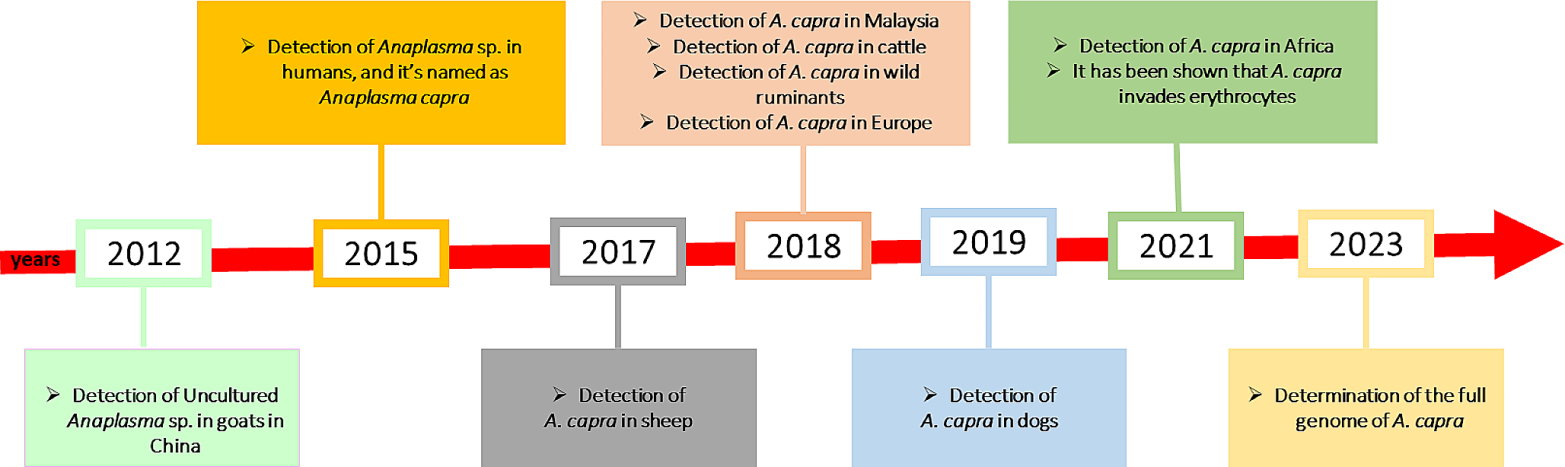
The cornerstones of *Anaplasma capra* history

The detection of the zoonotic potential in a short time (this period reached 60 years for *A. phagocytophilum*) resulted in the intense interest of the scientific community to *A. capra*. *Anaplasma phagocytophilum* was detected in sheep in 1932 (Gordon et al. 1932), but it took 62 years for it to be identified in humans (Chen et al. 1994). *A. capra* was detected for the first time in sheep and cattle in 2017 and 2018, respectively (Guo et al. 2018; Koh et al. 2018; Yang et al. 2017). In a study conducted in Malaysia in 2018, *A. capra* was detected for the first time in a country other than China (Koh et al. 2018). In the same year, *A. capra* was detected in takin, reeves’ muntjac, and forest musk deer, thus showing that it also infects wild animals (Yang et al. 2018). In 2018, *A. capra* was detected outside the Asian continent, with its discovery in Sweeden (Grandi et al. 2018). In 2019, the agent was detected in dogs in China. Thus, its presence in carnivores was determined for the first time (Shi et al. 2019). With the detection of *A. capra* in cattle from Angola in 2021, it was detected for the first time on the African continent (Barradas et al. 2021). It has been shown that *A. capra* invades host erythrocytes in 2021 (Peng et al. 2021a, b). Finally, in 2023, the full genome sequences of *A. capra* was obtained, and it was determined that the approximately 1.07 Mp genome contained 862 protein-coding genes (Lin et al. 2023a). It took a total of 12 years from the detection of *A. capra* to the disclosure of its full genome. The cornerstones of the short-fast historical process are summarized in Fig. 1.

## The hosts of *Anaplasma capra*

The life cycle of *Anaplasma* species circulates between vertebrate hosts and ticks (de la Fuente et al. 2016). The fact that *A. capra* was first detected in goats and then revealed in humans can be considered an indication that the host group will be interesting.

### Vertebrates

**Fig. 2:**
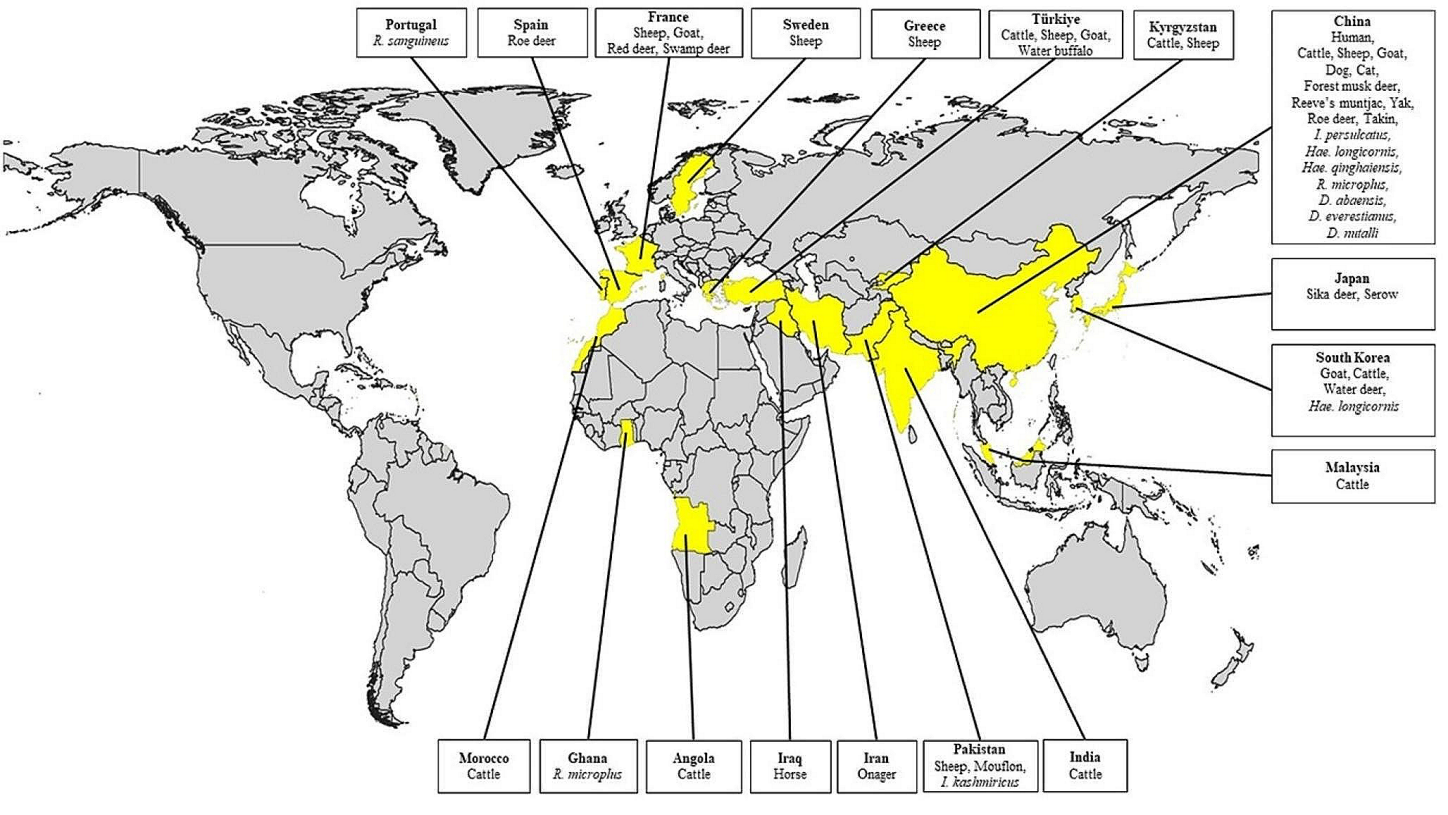
The countries and hosts (domestic animals, wild animals and ticks) which *Anaplasma capra* has been detected

The domestic and wild animals in which *A. capra* was detected by molecular methods are given in Fig. 2. Studies on *A. capra* using molecular methods were, as expected, mostly conducted on goats. This was followed by sheep and cattle. The study in China continued to be the first and only study in which *A. capra* was detected in humans (Li et al. 2015). While Li et al. (2015) reported that human-derived *A. capra* was demonstrated to infect HL-60 and THP-1 cells, Peng et al. (2021b) reported that the goat-derived *A. capra* can infect human erythrocytes, HL-60 and TF-1 cells as in vitro. The results can be considered as strong evidence for the zoonotic potential of *A. capra*. Except for humans, cattle, sheep, and goats, *A. capra* was detected in domestic animals such as buffalo (Sahin et al. 2022), dog (Shi et al. 2019), horse (unpublished data, GenBank; ON872236), cat (unpublished data, GenBank; MW520360), and wild animals such as roe deer (Remesar et al. 2022; Wang et al. 2019), sika deer (Kawahara et al. 2006), water deer (Shin et al. 2020), red deer (Jouglin et al. 2019), swamp deer (Jouglin et al. 2019), forest musk deer (Yang et al. 2018), yak (Wang et al. 2021b), onegar (Staji et al. 2021), serow (Sato et al. 2009), takin (Yang et al. 2018), mouflon (Isaq et al. 2022), and reeves’ muntjac (Yang et al. 2018), albeit in one or at most two studies (Fig. 2). Although some studies state that the main host of *A. capra* may be domestic ruminants, it seems that it is too early to say this. As a matter of fact, studies in this field were mostly conducted on domestic ruminants.

It is known that wild animals, especially wild ruminants such as red deer (*Cervus elaphus*), roe deer (*Capreolus capreolus*), white-tailed deer (*Odocoileus virginianus*), mouflon (*Ovis musimon*), and chamois (*Rupicapra rupicapra*), serve as reservoir hosts for *Anaplasma* species (Rar and Golovljova 2011). Among the animals in which *A. capra* has been detected, it is seen that wild ruminants are predominant (Fig. 2). Wild ruminates play an important role in the bioecology of *Anaplasma* species (Woldehiwet 2010). *Anaplasma ovis* has a high positivity rate in roe deer and red deer, and these species are reservoirs for *A. ovis* (de la Fuente et al. 2008; Renneker et al. 2013). A similar relationship is observed in *A. phagocytophilum* and deers (Teodorowski et al. 2020). The prevalence of *A. phagocytophilum* is up to 98% in roe deer and 87% in red deer (Stuen et al. 2013). It has also been reported that *A. marginale* is persistent in deer and has a high positivity rate in these animals (Atif 2016). Understanding the reservoir role of wild ruminants for *Anaplasma* species is important to explain the epidemiology of the species. Although de la Fourniere et al. (2023) recently showed transovarial transmission of *A. marginale* in *Rhipicephalus microplus* and Baldridge et al. (2009) demonstrated transovarial transmission of *A. phagocytophilum* in *Dermacentor albipictus*, it was generally thought that *Anaplasma* species were not transovarially transmitted in ticks (Aubry and Geale 2011; Kocan et al. 2010; Woldehiwet 2010). In this case, the reservoir role of wild animals increases their contribution to the epidemiology of *A. capra.*

### Ticks

Ticks are the main vector for numerous haemopathogens such as *Anaplasma, Babesia, Theileria*, and *Hepatozoon* (Dumanli et al. 2012; Inci et al. 2016). *Anaplasma* species are transmitted to hosts biologically through ticks belonging to the Ixodidae family, they are also transmitted mechanically through surgical instruments contaminated with the blood of infected animals, during surgical operations (such as castration or dehorning), through blood-sucking arthropods, or even transplacental (Aubry and Geale 2011; Dumler et al. 2001; Kocan et al. 2010). Many issues need to be clarified regarding the biology of *A. capra*, but its detection in blood-sucking arthropods is an indication of indirect development. The significantly higher prevalence of the agent in the summer months may be associated with vector activity (Shi et al. 2019; Wang et al. 2021b). The tick species in which *A. capra* was detected by molecular methods are given in Fig. 2. *Haemaphysalis longicornis* is the tick species on which the most studies have been conducted and for which *A. capra* has been detected the most (Guo et al. 2018; Lu et al. 2023; Qin et al. 2018; Seo et al. 2020; Sun et al. 2015; Teng et al. 2023; Yan et al. 2021). In much more limited work, *A. capra* was detected in *H. ginghainensis* (Han et al. 2019; Yang et al. 2016), *Ixodes persulcatus* (Li et al. 2015), I. *kashmiricus* (Numan et al. 2023), *Rhipicephalus sanguineus* (unpublished data, GenBank; OK091153), *R. microplus (*Addo et al. 2023; Guo et al. 2018, 2019) *Dermacontor nuttali, D. everestianus*, and *D. abaensis* (Han et al. 2019).

*Anaplasma capra* has been identified in both parasitic ticks (*H. longicornis*, *R. microplus, I. kashmiricus*) collected from domestic animals (Guo et al. 2018; Numan et al. 2023) and host-seeking (*H. longicornis, H. qinghaiensis, I. persulcatus, D. abaensis, D. everestianus, D. nuttalli*) ticks (Han et al. 2019; Li et al. 2015; Seo et al. 2020). The positivity rate in ticks is quite variable. It was detected in *I. persulcatus* at 3.0% (Li et al. 2015), in *H. longicornis* at 0.43–63.27% (Guo et al. 2018; Lu et al. 2023; Seo et al. 2020; Sun et al. 2015; Teng et al. 2023; Yan et al. 2021;), in *H. qinghaiensis* at 4.5–5.8% (Han et al. 2019; Yang et al. 2016), in *R. microplu*s at 0.81–40.4% positive rates (Addo et al. 2023; Guo et al. 2018, 2019). Almost all of the studies on ticks were conducted in China, where *A. capra* was first detected and where most studies were conducted on vertebrates. A very high positivity rate (63.27%) was determined in *H. longicornis* collected from goats in China (Lu et al. 2023). On the other hand, *H. longicornis* is the most prevalent tick species in China and is especially parasitized in sheep (Teng et al. 2023). It may support the relationship between *A. capra* infections and both small ruminants and *H. longicornis*. Although the current studies have revealed the presence of *A. capra* in ticks, there is a need to be further studies by transmission experiments. *Anaplasma marginale* is known to be transmitted by more than 20 tick species, including *D. andersoni, D. variabilis, D. albipictus, R. microplus*, and *R. annulatus* (Ben Said et al. 2018; Kocan et al. 2010). *Anaplasma capra* is likely to be identified in many more tick species. It has been detected in *R. sanguineus* from Portugal (unpublished data, GenBank; OK091153) and *I. kashmiricus* from Pakistan (Numan et al. 2023). Additionally, in two studies conducted in the same region (Sivas) in Türkiye, *A. capra* was detected at a rate of 14.28% in buffalos and 0.41% in cattle (Altay et al. 2022a; Sahin et al. 2022). The tick infestation rate is much lower in water buffalos than in cattle. However, the above prevalence contradicts this. It should be taken into consideration that *A. capra* can be transmitted by other means. It is known that some *Anaplasma* species are transmitted by other blood-sucking arthropods (Aubry and Geale 2011; Dumler et al. 2001; Kocan et al. 2010). Recently, *Ehrlichia* and *Rickettsia* species were detected in all developmental stages of mosquitoes, and it was reported that mosquitoes may transmit these species both transtadially and transovarially (Guo et al. 2016). When all this information is evaluated together, it shows that *A. capra* circulates among domestic animals, wild animals, and ticks, and that these hosts are important factors determining the epidemiology of *A. capra*.

## The molecular prevalence of *Anaplasma capra*

Although the studies on *A. capra* were mostly conducted in China, in a short time its presence has been revealed in 18 different countries including China (Liu et al. 2012), South Korea (Seo et al. 2018), Türkiye (Altay et al. 2022a), Kyrgyztan (Altay et al. 2022b, c), Malaysia (Koh et al. 2018), Japan (Kawahara et al. 2006), Iraq (unpublished data GenBank; ON872236), Iran (Staji et al. 2021), India (Kumar et al. 2023), Pakistan (Isaq et al. 2022), France (Jouglin et al. 2019), Sweeden (Grandi et al. 2018), Portugal (unpublished data, GenBank; OK091153), Spain (Remesar et al. 2022), Greece (Saratsis et al. 2022), Angola (Barradas et al. 2021), Morocco (Elhachimi et al. 2021), and Ghana (Addo et al. 2023) by molecular techniques (Fig. 1). Since, *A. capra* is a newly discovered species, studies have generally focused on its identification and determination of its phylogenetic position.

​The prevalence of tick-borne pathogens is affected by many different factors such as the host, age, season, management systems, tick infestation density, climatic characteristics of the region, host immunity, time of sampling, sample size, and detection methods (Belkahia et al. 2017; Ben Said et al. 2018; Kabir et al. 2011; Nguyen et al. 2020; Wang et al. 2021a). According to a meta-analysis study conducted in 2023, the average prevalence of *A. capra* was found to be 5.9% in humans, 11.3% in animals, and 7.8% in ticks (Lin et al. 2023b). Despite this undoubtedly valuable information, it is still very difficult to determine the limits of the prevalence of *A. capra*. The positivity rate of *A. capra* in cattle was 0.28% in Kyrgyzstan (Altay et al. 2022c), 0.30% in South Korea (Miranda et al. 2021), 0.41% in Türkiye (Altay et al. 2022a), and 11.3% in Morocco (Elhachimi et al. 2021). Similarly, while the positivity rate in goats is 0.30% in South Korea (Miranda et al. 2021), this rate reaches 44.6% in China (Wei et al. 2020). Its prevalence in wild animals starts from 0.6% (Wang et al. 2021b) and reaches 17.7% (Amer et al. 2019). The positivity rates obtained from molecular studies conducted on domestic and wild animals can be viewed in Table 1.

**Table 1:** Molecular prevalence studies of *Anaplasma capra* in domestic and wild animals

Country	Host	Number of samples	Positivity rate (%)	References
**China**	goat	731	3.4	Zhou et al. 2023
		943	9.4	Peng et al. 2018
		174	9.8	Guo et al. 2018
		357	12.3	Yang et al. 2017
		491	26.6	Wang et al. 2021a
		92	44.6	Wei et al. 2020
		72	59.7	Lin et al. 2023a
	sheep	510	7.8	Peng et al. 2018
		341	10.0	Shi et al. 2020
		95	10.5	Guo et al. 2018
		190	16.3	Yang et al. 2017
		435	18.2	Yang et al. 2018
		18	53.7	Lin et al., 2023
		309	56.0	He et al. 2021
	cattle	36	5.6	Guo et al. 2018
	dog	521	12.1	Shi et al. 2019
	roe deer	9	33.3	Wang et al. 2019
	forest musk deer	s1	100	Yang et al. 2018
	reeve’s muntjac	3	66.7	Yang et al. 2018
	takin	5	60.0	Yang et al. 2018
	yak	330	0.6	Wang et al. 2021b
**South Korea**	cattle	384	0.3	Miranda et al. 2021
		1,219	0.4	Seo et al. 2018*
	goat	302	0.3	Miranda et al. 2021
	water deer	28	14.3	Shin et al. 2020
		198	17.7	Amer et al. 2019
**Türkiye**	cattle	241	0.41	Altay et al. 2022a
	goat	200	0.5	Oguz et al. 2023
	sheep	155	3.22	Altay et al. 2022a
	water buffalo	364	14.28	Sahin et al. 2022
**Kyrgyzstan**	sheep	391	5.3	Altay et al. 2022b
	cattle	358	0.28	Altay et al. 2022c
**Malaysia**	cattle	224	1.4	Koh et al. 2019
**Pakistan**	sheep	105	29.52	Ishaq et al. 2022
	mouflon (wild sheep)	105	11.43	Ishaq et al. 2022
**Iran**	onegar	20	20.0	Staji et al. 2021
**France**	sheep	70	30.0	Jouglin et al. 2022
	goat	38	13.2	Jouglin et al.2022
	red deer	59	3.4	Jouglin et al. 2019
	swamp deer	7	14.3	Jouglin et al. 2019
**Spain**	roe deer	224	5.8	Remesar et al. 2022**
**Morocco**	cattle	257	11.3	Elhachimi et al. 2021
**Angola**	cattle	98	6.12	Barradas et al. 2021

Tick activation is generally highest between spring and autumn (Dumanli et al. 2012). The prevalence of *A. capra* was found to be higher in the summer months, which are more suitable for the activation of ticks, as in other tick-borne pathogens (Seo et al. 2018; Shi et al. 2019). Additionally, it was observed that its prevalence increased with age, and in this case, it was associated with the extension of the tick contact period (Shi et al. 2019; Zhou et al. 2023). It should be taken into consideration that *A. capra* may be chronic or persistent and its prevalence may increase with age (Rar et al. 2021). Jouglin et al. (2019) reported that *A. capra* can persist in red deer for four months. The persistently infected hosts may serve as reservoirs for ticks, and these hosts are important in the epidemiology of the *Anaplasm*a species (Brown and Barbet 2016; Kocan et al. 2010). Although the prevalence of *A. capra* varies, the important point is that its circulation is in a wide geography and a very wide host group. Considering the current prevalence of the agent and the fact that it has been identified in different tick species, studies are needed to determine its situation in other continents.

## The genotypes of *Anaplasma capra*

In recent years, intensive studies have been carried out on the genetic differences of *Anaplasma* species and the relationship of some genetic groups with geography, vector and host is emphasized (Rar et al. 2021). In the multilocus sequence analysis of 520 samples of *A. phagocytophilum*, eight clusters that could be separated according to geography, vector, and host were obtained (Langenwalder et al. 2020). Twelve clusters emerged in phylogenetic analyses based on the *A. phagocytophilum ankA* gene (Langenwalder et al. 2020). In the analysis of *groEL* sequences of *A. phagocytophilum* from Europe and Russia, four different ecotypes with host tropism were identified (Jahfari et al. 2014). The 11 5’-UTR microsatellite genotypes and 193 *msp1a* tandem repeats of *A. marginale* have been identified worldwide, but it has been reported that they have no geographical relationship (Rar et al. 2021). The 47 *msp1aS* repeats and 32 genotypes of *A. centrale* have been identified only in Africa (Khumalo et al. 2018). *msp2* gene analyses of *A. ovis* have revealed between 2 and 17 genotypes in different countries (Belkahia et al. 2014, 2017; Cabezas-Cruz et al. 2019; Torina et al. 2010; Zhou et al. 2017). *Anaplasma bovis groEL* sequences form four and *gltA* sequences form three lineages. There are findings that ecotypes formed on this basis show host and vector specificity (Rar et al. 2021).

The *16S rRNA* gene is frequently used in molecular survey studies. However, *16S rRNA* gene is not very useful in *Anaplasma* species genotyping studies, more variable genes (*groEL, gltA, msp2*, and *msp4*) are preferred in these studies (Caudill and Brayton 2022; Rar et al. 2021). After the naming of *A. capra* in 2015, especially the *groEL* and *gltA* gene sequences were recorded in GenBank (NCBI). Thus, a sequence pool of this isolate was formed, which enabled intraspecific genetic comparisons. Yang et al. (2017) reported that the *16S rRNA* gene of *A. capra* exhibits high sequence similarity (similarity of 99.8–99.9%), but the *gltA* and *groEL* genes were relatively less identical (88.6–88.7% for *gltA* and 90.6–91.0% for *groEL*). They concluded that; one genotype contains strains isolated from goats, sheep, *I. persulcatus*, and humans, while the other from deer, serows, and *H. qinghaiensis*. Wang et al. (2021a) similarly reported that *A. capra* was divided into two clusters, and cluster I contained isolates with zoonotic potential (from human), and clade II contained isolates obtained from goats. However, it has been reported that *A. capra* exhibits at least two different genotypes, both are likely zoonotic (Peng et al. 2021a). Jouglin et al. (2022) reported that *A. capra* divides in two separate clades based on *gltA* or *groEL*, clade I includes *A. capra* sequences from sheep, goats, cattle, dogs, humans, and ticks, and clade II includes from sheep and goats, and also from a variety of wild ruminants. They reported that this grouping had no geographical relationship (Jouglin et al. 2022). In the analysis of 203 *gltA* gene sequences, water buffalo, sheep, goat, wild animals, and tick isolates were included in the genotype-1 group, and human, sheep, cattle, goat, dog, wild animals, and tick isolates were in the genotype-2 group. In the analysis of 158 *groEL* gene sequences, water buffalo, sheep, goat, wild animals, and tick isolates were included in genotype-1, and human, dog, cat, sheep, goats, cattle, wild animals, and tick isolates were included in genotype-2 (Sahin et al. 2022). When the *gltA* DNA sequences of 21 *A. capra* isolates detected in Kyrgyzstan in 2022 were compared with the existing gene sequences in the Gene Bank, one group with a difference of 0–7 nucleotides within themselves and the second group with a difference of 68–70 nucleotides from those were formed. The first group includes the sequences from red deer, swamp deer, Siberian roe deer, takin, reeves’ muntjac, forest musk deer, *D. everestianus*, Korean water deer, cattle, and sheep, while the second group includes dog, cattle, sheep, goat, human, *H. qinghaiensis, H. longicornis*, and *R. microplus* (Altay et al. 2022b). In another study conducted on the basis of *gltA*, isolates from France (red deer, swamp deer), South Korea (water deer), China (*D. everestianus*), Türkiye (cattle and sheep) were classified in the genotype-1 group, China (human, dog, sheep, goat and *R. microplus*) and South Korea (cattle) were included in the genotype-2 group (Altay et al. 2022a). *Anaplasma capra* isolates obtained from sheep in Kyrgyzstan are in the genotype-1 group with isolates from France (swamp deer and red deer), and China (roe deer, takin, forest musk deer, revees’ muntjac and *D. everestianus*) but China (human, goat, sheep, dog, *H. longicornis*, *H. qinghaiensis*, and *R. microplus*) isolates were included in the genotype-2 group (Altay et al. 2022b). According to these data, it can be thought that human isolates are genotype-2 and while only genotype-1 is found in Europe, both genotypes are found in Asia. This situation may change with the addition of more sequences from different isolates. There is a need for comprehensive and detailed research to reveal the relationship between host, tick species, geography, and pathogenicity of these isolates.

**Fig. 3:**
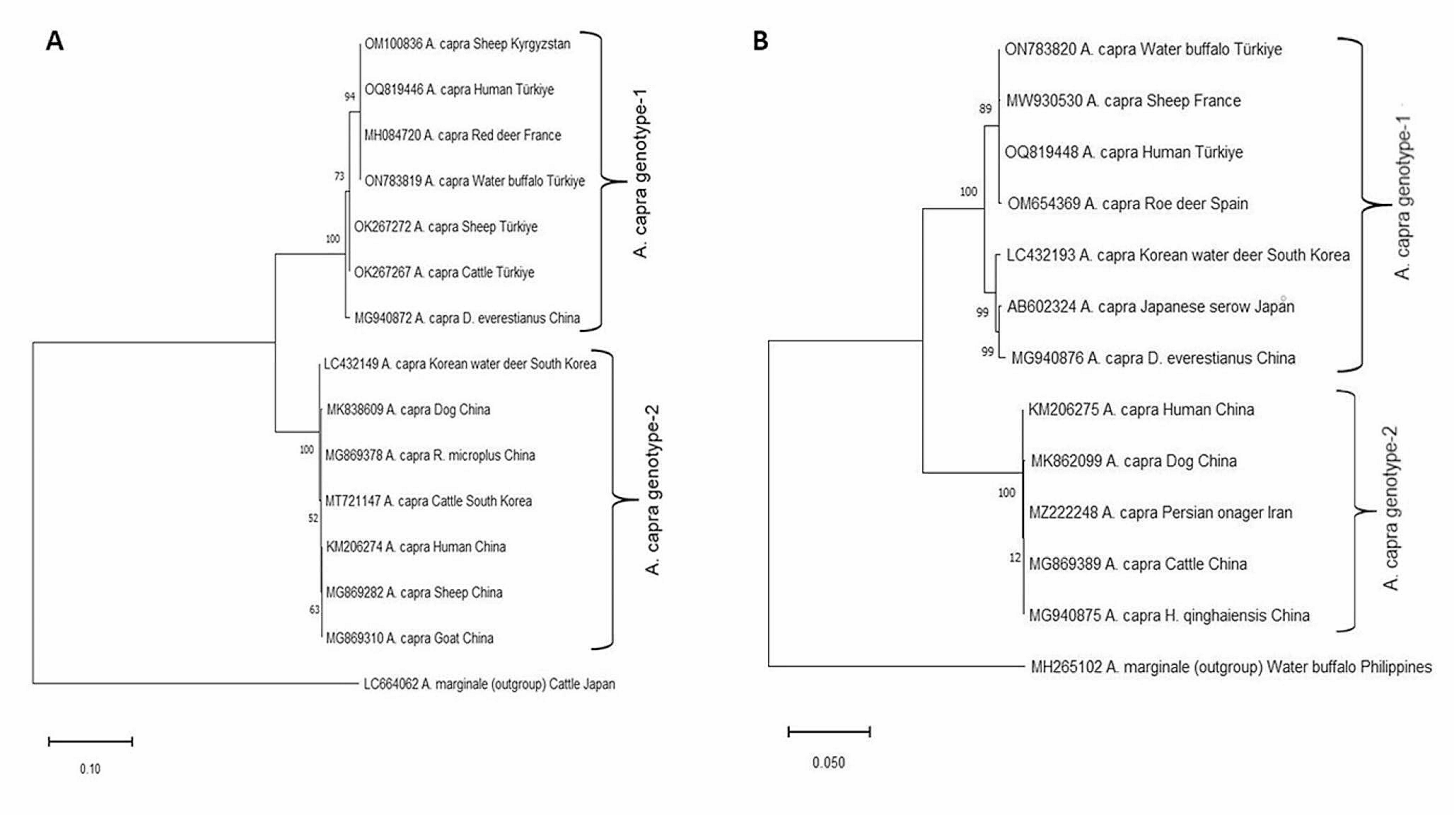
Phylogenetic tree of *Anaplasma capra* genotypes. (A) *gltA* gene region, (B) *groEL* gene region

In this review, a phylogenetic tree based on *gltA* and *groEL* genes, which are more informative to understand genotypic variation of the pathogen than other gene regions, like *16S rRNA* was constructed by selecting some of the *A. capra* isolates detected in different parts of the world. In this phylogenetic tree, *A. capra* genotype-1 and *A. capra* genotype-2 are shown (Fig. 3).

## Pathogenicity and public health concern of *Anaplasma capra*

*Anaplasma* species infect different cells of the host and multiply within these cells. While *A. ovis, A. marginale*, and *A. centrale* infect the erythrocytes, *A. bovis, A. phagocytophilum*, and *A. platys* infect monocytes, granulocytes, and platelets, respectively (Dumler et al. 2001; Kocan et al. 2010; Woldehiwet 2010). While the morulae of *A. marginale* are small and localized at the periphery of stained erythrocytes, *A. centrale* forms smaller and more central morulae (Theiler 1910). *Anaplasma ovis* settles in the central area of sheep erythrocytes (Bevan 1912). Current information shows that *A. capra* invades erythrocytes like *A. marginale, A. centrale*, and *A. ovis* (Peng et al. 2021b). Li et al. (2015) inoculated *A. capra* into human cell lines (HL-60 and THP-1 cells) and observed the morulae stages. However, in natural infections, no morulae or other blood agents were detected in any cells of peripheral blood smears by microscopic examination. (Li et al. 2015). *Anaplasma capra* inclusion bodies were microscopically observed in the erythrocytes of naturally infected onagers, but their morphological features were not defined (Staji et al. 2021). The first detailed study on the morphology of *A. capra* was carried out by Peng et al. (2021a). The researchers examined *A. capra*-infected goat erythrocytes using electron microscope. They observed *A. capra* cells with a diameter of 0.2–0.4 μm on the outer surface of the erythrocyte. They also imaged the round or oval-shaped morulae stages, on the surface (0.2–0.4 μm in diameter) and inside (0.8 × 1 μm) of the erythrocytes. Peng et al. (2021b) showed that goat-derived *A. capra* can infect human erythrocytes and TF-1 cells.

The clinical course of anaplasmosis varies depending on the species causing the disease. *Anaplasma phagocytophilum* is the causative agent of tick fever syndrome in ruminants and causes high fever, loss of appetite, cough, decrease in milk yield, and abortions (Woldehiwet 2010). *Anaplasma marginale* causes severe clinical symptoms in cattle (Kocan et al. 2010) and can cause anemia, weakness, fever, loss of appetite, decrease in milk yield, abortion, and death in untreated cases (Aubry and Geale 2011; Kocan et al. 2010). *Anaplasma bovis* can cause fever, weight loss, incoordination, lymph node enlargement, and rarely death in ruminants (Chilton et al. 2018). *Anaplasma centrale* is less pathogenic than *A. marginale* and usually causes subclinical infections in cattle, it is used as live vaccines against anaplasmosis caused by *A. marginale* in Israel, South Africa, South America, and Australia (Aubry and Geale 2011; Kolo 2023). While *A. ovis* generally causes mild clinical symptoms in sheep and goats, it can also cause acute infections with clinical symptoms such as hemolytic anemia, icterus, depression, anorexia, weight loss, and decreased milk yield in case the immune system of infected hosts is suppressed or in mixed infections with different pathogens (Dumler et al. 2001; Renneker et al. 2013). While *A. platys* causes infectious cyclic thrombocytopenia in dogs, there are usually no symptoms of infection (de la Fuente et al. 2006).

There is not yet sufficient information that *A. capra* causes clinical disease in animals. The prevalence of the agent was found only to be high in the anemia group in dogs (Shi et al. 2019). However, in the same study, no relationship was found between *A. capra* and fever, cough, malaise, and depression (Shi et al. 2019). Current information shows that *A. capra* invades host erythrocytes, such as *A. marginale, A. centrale*, and *A. ovis* (Peng et al. 2021a, b). Staji et al. (2021) reported that a slight decrease of RBC, HCT, and HGB, leukopenia, lymphopenia, thrombocytopenia, hypoalbuminemia, and hyperbilirubinemia was detected in two Persian onagers infected with *A. capra*. The clinical picture of human infections is relatively clearer. It has been reported that *A. capra* causes influenza-like symptoms, including fever, headache, malaise, dizziness, and chills, and some gastrointestinal symptoms (nausea, vomiting, or diarrhea), rash, eschar, and regional lymphadenopathy in humans. Additionally, high hepatic aminotransferase concentrations, leucopenia, and thrombocytopenia have been detected. The patients recovered when treated with doxycycline (Li et al. 2015).

## Conclusions

Tick-borne pathogens are a growing concern for human and animal health. *Anaplasma capra* has been detected in humans, domestic animals, wild animals, and ticks. Although current studies are not sufficient, the domestic ruminants are considered the main host. Wild animals serve as reservoirs for many tick-borne pathogens. *Anaplasma capra* has been detected in various wild animal species. The existence of clinical infections in humans has been demonstrated, although in limited studies. The detection of the agent in parasitic and host-seeking ticks indicates that it is a tick-borne disease. All the studies together show that *A. capra* has a wide host range with an increased risk of infections worldwide. Considering that the clinical symptoms of anaplasmosis infections are significantly nonspecific and are confused with other infections, the importance of *A. capra* to human health can be better emphasized. *Anaplasma capra*, as an emerging bacterial infection transmitted by ticks, with global distribution, and a very wide host group, is a species that needs to be investigated in many aspects, especially its epidemiology, biological properties, pathogenicity via experimental infections in different hosts, rapid diagnosis, and, if necessary, treatment protocols and effective control methods.

## Data Availability

No datasets were generated or analysed during the current study.

## References

[CR1] Addo SO, Baako BOA, Bentil RE, Addae CA, Behene E, Asoala V, Sallam M, Mate S, Dunford JC, Larbi JA, Baidoo PK, Wilson MD, Diclaro JW, Dadzie SK (2023). Molecular survey of *Anaplasma* and *Ehrlichia* species in livestock ticks from Kassena-Nankana, Ghana; with a first report of *Anaplasma Capra* and *Ehrlichia Minasensis*. Arch Microbiol.

[CR2] Altay K, Erol U, Sahin OF (2022). The first molecular detection of *Anaplasma Capra* in domestic ruminants in the central part of Turkey, with genetic diversity and genotyping of *Anaplasma Capra*. Trop Anim Health Prod.

[CR3] Altay K, Erol U, Sahin OF, Aytmirzakizi A, Temizel EM, Aydin MF, Dumanli N, Aktas M (2022). The detection and phylogenetic analysis of *Anaplasma phagocytophilum*-like 1, *A*. *ovis* and *A*. *capra* in sheep: *A*. *capra* divides into two genogroups. Vet Res Commun.

[CR4] Altay K, Erol U, Sahin OF, Aytmirzakızı A (2022). First molecular detection of *Anaplasma* species in cattle from Kyrgyzstan; molecular identification of human pathogenic novel genotype *Anaplasma Capra* and *Anaplasma phagocytophilum* related strain. Ticks Tick Borne Dis.

[CR5] Amer S, Kim S, Yun Y, Na KJ (2019). Novel variants of the newly emerged *Anaplasma Capra* from Korean water deer (*Hydropotes inermis argyropus*) in South Korea. Parasit Vector.

[CR6] Atif FA (2016). Alpha proteobacteria of genus *Anaplasma* (Rickettsiales: Anaplasmataceae): epidemiology and characteristics of *Anaplasma* species related to veterinary and public health importance. Parasitology.

[CR7] Aubry P, Geale DW (2011). A review of bovine anaplasmosis. Transbound Emerg Dis.

[CR8] Baldridge GD, Scoles GA, Burkhardt NY, Schloeder B, Kurtti TJ, Munderloh UG (2009). Transovarial transmission of *Francisella*-like endosymbionts and *Anaplasma phagocytophilum* variants in *Dermacentor albipictus* (Acari: Ixodidae). J Med Entomol.

[CR9] Barradas PF, Mesquita JR, Ferreira P, Gartner F, Carvalho M, Inacio E, Chivinda E, Katimba A, Amorim I (2021). Molecular identification and characterization of *Rickettsia* spp. and other tick-borne pathogens in cattle and their ticks from Huambo, Angola. Tick Tick Borne Dis.

[CR11] Belkahia H, Ben Said M, El Hamdi S, Yahiaoui M, Gharbi M, Daaloul-Jedidi M, Messadi L (2014). First molecular identification and genetic characterization of *Anaplasma ovis* in sheep from Tunisia. Small Rum Res.

[CR10] Belkahia H, Ben Said M, El Mabrouk N, Saidani M, Cherni C, Ben Hassen M, Bouattour A, Messadi L (2017). Seasonal dynamics, spatial distribution and genetic analysis of *Anaplasma* species infecting small ruminants from Northern Tunisia. Infect Genet Evol.

[CR12] Ben Said M, Belkahia H, Messadi L (2018). *Anaplasma* spp. in North Africa: a review on molecular epidemiology, associated risk factors and genetic characteristics. Ticks Tick Borne Dis.

[CR13] Bevan LLEW (1912). Anaplasmosis of sheep. Vet J.

[CR14] Brown WC, Barbet AF (2016). Persistent infections and immunity in ruminants to arthropod-borne bacteria in the family Anaplasmataceae. Annu Rev Anim Biosci.

[CR16] Cabezas-Cruz A, Gallois M, Fontugne M, Allain E, Denoual M, Moutailler S, Devillers E, Zientara S, Memmi M, Chauvin A, Agoulon A, Vayssier Taussat M, Chartier C (2019) Epidemiology and genetic diversity of *Anaplasma ovis* in goats in Corsica, France. Parasit Vector 12: 3. 10.1186/s13071-018-3269-7 10.1186/s13071-018-3269-7 PMC6318933 30606253

[CR15] Caudill MT, Brayton KA (2022). The use and limitations of the *16S rRNA* sequence for species classification of *Anaplasma* samples. Microorganisms.

[CR17] Chen SM, Dumler JS, Bakken JS, Walker DH (1994). Identification of a granulocytic *Ehrlichia* species as the etiologic agent of human disease. J Clin Microbiol.

[CR18] Chilton NB, Dergousoff SJ, Lysyk TJ (2018). Prevalence of *Anaplasma bovis* in Canadian populations of the Rocky Mountain wood tick, *Dermacentor andersoni*. Ticks Tick Borne Dis.

[CR19] de la Fourniere S, Guillemi EC, Paoletta MS, Pérez A, Obregón D, Cabezas-Cruz A, Sarmiento NF, Farber MD (2023). Transovarial transmission of *Anaplasma marginale* in *Rhipicephalus* (*Boophilus*) *microplus* ticks results in a bottleneck for. Strain Divers Pathogens.

[CR20] de la Fuente J, Torina A, Naranjo V, Nicosia S, Alongi A, La Mantia F, Kocan KM (2006). Molecular characterization of *Anaplasma platys* strains from dogs in Sicily, Italy. BMC Vet Res.

[CR22] de la Fuente J, Ruiz Fons F, Naranjo V, Torina A, Rodriguez O, Gortazar C (2008). Evidence of *Anaplasma* infections in European roe deer (*Capreolus capreolus*) from southern Spain. Res Vet Sci.

[CR21] de la Fuente J, Estrada-Pena A, Cabezas Cruz A, Kocan KM (2016). *Anaplasma phagocytophilum* uses common strategies for infection of ticks and vertebrate hosts. Trends Microbiol.

[CR23] Dumanli N, Altay K, Aydin MF (2012). Tick species of cattle, sheep and goats in Turkey. Turkiye Klinikleri J Vet Sci.

[CR24] Dumler JS, Barbet AF, Bekker CP, Dasch GA, Palmer GH, Ray SC, Rikihisa Y, Rurangirwa FR (2001). Reorganization of genera in the families Rickettsiaceae and Anaplasmataceae in the order Rickettsiales: unification of some species of *Ehrlichia* with *Anaplasma*, *Cowdria* with *Ehrlichia* and *Ehrlichia* with *Neorickettsia*, descriptions of six new species combinations and designation of *Ehrlichia equi* and ‘HGE agent’ as subjective synonyms of *Ehrlichia phagocytophila*. Int J Syst Evol Microbiol.

[CR25] Elhachimi L, Rogiers C, Casaert S, Fellahi S, Van Leeuwen T, Dermauw W, Valcarcel F, Olmeda AS, Daminet S, Khatat SEH, Sahibi H, Duchateau L (2021). Ticks and tick-borne pathogens abound in the cattle population of the Rabat-Sale Kenitra Region. Morocco Pathogens.

[CR26] Gordon WS, Brownlee A, Wilson DR, MacLeod J (1932). Tick-borne fever (a hitherto undescribed disease of sheep). J Comp Pathol.

[CR27] Grandi G, Aspan A, Pihl J, Gustafsson K, Engstrom F, Jinnerot T, Soderlund R, Chirico J (2018). Detection of tick-borne pathogens in lambs undergoing prophylactic treatment against ticks on two Swedish farms. Front Vet Sci.

[CR28] Guo WP, Tian JH, Lin XD, Ni XB, Chen XP, Liao Y, Yang SY, Dumler JS, Holmes EC, Zhang YZ (2016). Extensive genetic diversity of Rickettsiales bacteria in multiple mosquito species. Sci Rep.

[CR29] Guo WP, Huang B, Zhao Q, Xu G, Liu B, Wang YH, Zhou EM (2018) Human-pathogenic *Anaplasma* spp., and *Rickettsia* spp. in animals in Xi’an, China. PLoS Negl Trop Dis 12(11): e0006916. 10.1371/journal.pntd.0006916 10.1371/journal.pntd.0006916 PMC6258427 30419024

[CR30] Guo WP, Zhang B, Wang YH, Xu G, Wang X, Ni X, Zhou EM (2019). Molecular identification and characterization of *Anaplasma Capra* and *Anaplasma platys*-like in *Rhipicephalus microplus* in Ankang, Northwest China. BMC Infect Dis.

[CR31] Han R, Yang JF, Mukhtar MU, Chen Z, Niu QL, Lin YQ, Liu GY, Luo JX, Yin H, Liu ZJ (2019). Molecular detection of *Anaplasma* infections in ixodid ticks from the Qinghai-Tibet Plateau. Infect Dis Poverty.

[CR32] He Y, Chen W, Ma P, Wei Y, Li R, Chen Z, Tian S, Qi T, Yang J, Sun Y, Li J, Kang M, Li Y (2021). Molecular detection of Anaplasma spp., Babesia spp. and Theileria spp. in yaks (Bos grunniens) and tibetan sheep (Ovis aries) on the Qinghai-Tibetan Plateau, China. Parasit Vector.

[CR33] Inci A, Yildirim A, Duzlu O, Doganay M, Aksoy S (2016). Tick-borne diseases in Turkey: a review based on one health perspective. PLoS Negl Trop Dis.

[CR34] Inokuma H, Terada Y, Kamio T, Raoult D, Brouqui P (2001). Analysis of the *16S rRNA* gene sequence of *Anaplasma centrale* and its phylogenetic relatedness to other ehrlichiae. Clin Diagn Lab Immunol.

[CR35] Ishaq M, Ijaz M, Lateef M, Ahmed A, Muzammil I, Javed MU, Raza A, Ghumman NZ (2022). Molecular characterization of *Anaplasma Capra* infecting captive mouflon (*Ovis Gmelini*) and domestic sheep (*Ovis aries*) of Pakistan. Small Rum Res.

[CR36] Jahfari S, Coipan EC, Fonville M, van Leeuwen AD, Hengeveld P, Heylen D, Heyman P, van Maanen C, Butler CM, Foldvari G, Szekeres S, van Duijvendijk G, Tack W, Rijks JM, van der Giessen J, Takken W, van Wieren SE, Takumi K, Sprong H (2014). Circulation of four *Anaplasma phagocytophilum* ecotypes in Europe. Parasit Vector.

[CR37] Jouglin M, Blanc B, de la Cotte N, Bastian S, Ortiz K, Malandrin L (2019). First detection and molecular identification of the zoonotic *Anaplasma capra* in deer in France. PLoS ONE.

[CR38] Jouglin M, Rispe C, Grech-Angelini S, Gallois M, Malandrin L (2022). *Anaplasma capra* in sheep and goats on Corsica Island, France: a European lineage within *A. capra* clade II?. Ticks Tick Borne Dis.

[CR39] Kabir MHB, Mondal MMH, Eliyas M, Mannan MA, Hashem MA, Debnath NC, Miazi OF, Mohiuddin C, Kashem MA, Islam MR, Elahi MF (2011). An epidemiological survey on investigation of tick infestation in cattle at Chittagong District, Bangladesh. Afr J Microbiol Res.

[CR40] Kawahara M, Rikihisa Y, Lin Q, Isogai E, Tahara K, Itagaki A, Hiramitsu Y, Tajima T (2006). Novel genetic variants of *Anaplasma phagocytophilum, Anaplasma bovis, Anaplasma centrale*, and a novel *Ehrlichia* sp. in wild deer and ticks on two major islands in Japan. Appl Environ Microbiol.

[CR41] Khumalo ZTH, Brayton KA, Collins NE, Chaisi ME, Oosthuizen MC (2018). Evidence confirming the phylogenetic position of *Anaplasma centrale* (ex Theiler 1911) Ristic and Kreier 1984. Inter J Syst Evol Microbiol.

[CR42] Kocan KM, de la Fuente J, Blouin EF, Coetzee JF, Ewing SA (2010). The natural history of *Anaplasma marginale*. Vet Parasitol.

[CR43] Koh FX, Panchadcharam C, Sitam FT, Tay ST (2018). Molecular investigation of *Anaplasma* spp. in domestic and wildlife animals in Peninsular Malaysia. Vet Parasitol Reg Stud Rep.

[CR44] Kolo A (2023). *Anaplasma* species in Africa - a century of discovery: a review on molecular epidemiology, genetic diversity, and control. Pathogens.

[CR45] Kumar K, Singh K, Verma AK, Maurya PS, Prajapati MR, Kumar A, Sarkar TK (2023). Phylogenetic analysis and molecular characterization of field isolates of *Anaplasma* spp. from cattle in India. Vet Arhiv.

[CR46] Langenwalder DB, Schmidt S, Silaghi C, Skuballa J, Pantchev N, Matei IA, Mihalca AD, Gilli U, Zajkowska J, Ganter M, Hoffman T, Salaneck E, Petrovec M, von Loewenich FD (2020). The absence of the drhm gene is not a marker for human-pathogenicity in European *Anaplasma phagocytophilum* strains. Parasit Vector.

[CR47] Li H, Zheng YC, Ma L, Jia N, Jiang BG, Jiang RR, Huo QB, Wang YW, Liu HB, Chu YL, Song YD, Yao NN, Sun T, Zeng FY, Dumler JS, Jiang JF, Cao WC (2015). Human infection with a novel tick-borne *Anaplasma* species in China: a surveillance study. Lancet Infect Dis.

[CR48] Lin ZT, Du LF, Zhang MZ, Han XY, Wang BH, Meng J, Yu FX, Zhou XQ, Wang N, Li C, Wang XY, Liu J, Gao WY, Ye RZ, Xia LY, Sun Y, Jia N, Jiang JF, Zhao L, Cui XM, Zhan L, Cao WC (2023). Genomic characceristics of emerging intraerythrocytic *Anaplasma capra* and high prevalence in goats, China. Emerg Infect Dis.

[CR49] Lin ZT, Ye RZ, Liu JY, Wang XY, Zhu WJ, Li YY, Cui XM, Cao WC (2023). Epidemiological and phylogenetic characteristics of emerging *Anaplasma capra*: a systematic review with modeling analysis. Infec Genet Evol.

[CR50] Liu Z, Ma M, Wang Z, Wang J, Peng Y, Li Y, Guan G, Luo J, Yin H (2012). Molecular survey and genetic identification of *Anaplasma* species in goats from central and southern China. Appl Environ Microbiol.

[CR51] Lu M, Meng C, Li Y, Zhou G, Wang L, Xu X, Li N, Ji Y, Tian J, Wang W, Li K (2023). Rickettsia sp. and Anaplasma spp. in Haemaphysalis longicornis from Shandong Province of China, with evidence of a novel species Candidatus Anaplasma shandongensis. Ticks Tick Borne Dis.

[CR52] Miranda EA, Han SW, Cho YK, Choi KS, Chae JS (2021). Co-infection with *Anaplasma* species and novel genetic variants detected in cattle and goats in the Republic of Korea. Pathogens.

[CR53] Nguyen AH, Tiawsirisup S, Kaewthamasorn M (2020). Molecular detection and genetic characterization of *Anaplasma marginale* and *Anaplasma platys*-like (Rickettsiales: Anaplasmataceae) in water buffalo from eight provinces of Thailand. BMC Vet Res.

[CR54] Numan M, Alouffi A, Almutairi MM, Tanaka T, Ahmed H, Akbar H, Rashid MI, Tsai KH, Ali A (2023). First detection of *Theileria sinensis*-like and *Anaplasma Capra* in *Ixodes kashmiricus*: with notes on *cox1*-based phylogenetic position and new locality records. Animals.

[CR55] Oguz B, Deger MS, Al Olayan E, El Ashram S (2023). Molecular survey of *Anaplasma Capra* in goats in Van province, eastern Türkiye. Acta Parasitol.

[CR58] Peng Y, Wang K, Zhao S, Yan Y, Wang H, Jing J, Jian F, Wang R, Zhang L, Ning C (2018). Detection and phylogenetic characterization of *Anaplasma Capra*: an emerging pathogen in sheep and goats in China. Front Cell Infect Microbiol.

[CR56] Peng Y, Lu C, Yan Y, Shi K, Chen Q, Zhao C, Wang R, Zhang L, Jian F, Ning C (2021). The first detection of *Anaplasma Capra*, an emerging zoonotic *Anaplasma* sp., in erythrocytes. Emerg Microbes Infect.

[CR57] Peng Y, Lu C, Yan Y, Song J, Pei Z, Gong P, Wang R, Zhang L, Jian F, Ning C (2021). The novel zoonotic pathogen, *Anaplasma Capra*, infects human erythrocytes, HL-60, and TF-1 cells in vitro. Pathogens.

[CR59] Qin XR, Han FJ, Luo LM, Zhao FM, Han HJ, Zhang ZT, Liu JW, Xue ZF, Liu MM, Ma DQ, Huang YT, Yue S, Sun XF, Li WQ, Zhao L, Hao Y, Yu XJ (2018). *Anaplasma* species detected in *Haemaphysalis longicornis* tick from China. Ticks Tick Borne Dis.

[CR60] Rar V, Golovljova I (2011). *Anaplasma, Ehrlichia*, and Candidatus Neoehrlichia bacteria: pathogenicity, biodiversity, and molecular genetic characteristics, a review. Infect Genet Evol.

[CR61] Rar V, Tkachev S, Tikunova N (2021). Genetic diversity of *Anaplasma* bacteria: twenty years later. Infect Genet Evol.

[CR62] Remesar S, Prieto A, Garcia-Dios D, Lopez-Lorenzo G, Martinez-Calabuig N, Diaz-Cao JM, Panadero R, Lopez CM, Fernandez G, Díez-Banos P, Morrondo P, Diaz P (2022). Diversity of *Anaplasma* species and importance of mixed infections in roe deer from Spain. Transbound Emerg Dis.

[CR63] Renneker S, Abdo J, Salih DE, Karagenç T, Bilgiç H, Torina A, Oliva AG, Campos J, Kullmann B, Ahmed J, Seitzer U (2013). Can *Anaplasma ovis* in small ruminants be neglected any longer?. Transbound Emerg Dis.

[CR64] Sahin OF, Erol U, Altay K (2022) Buffaloes as new hosts for *Anaplasma capra*: molecular prevalence and phylogeny based on *gtlA, groEL*, and *16S rRNA* genes. Res Vet Sci 152: 458–464. 10.1016/j.rvsc.2022.09.008 10.1016/j.rvsc.2022.09.008 36148715

[CR65] Saratsis A, Ligda P, Aal F, Jelicic M, Polgar J, de Vries M, Mastranestasis I, Musella V, Rinaldi L, Jongejan F, Sotiraki S (2022). The scenario of ticks and tick-borne pathogens of sheep on a Mediterranean Island. Microorganisms.

[CR66] Sato M, Nishizawa I, Fujihara M, Nishimura T, Matsubara K, Harasawa R (2009). Phylogenetic analysis of the 16S rRNA gene of *Anaplasma* species detected from Japanese serows (*Capricornis crispus*). J Vet Med Sci.

[CR68] Seo MG, Ouh IO, Lee H, Geraldino PJL, Rhee MH, Kwon OD, Kwak D (2018). Differential identification of *Anaplasma* in cattle and potential of cattle to serve as reservoirs of *Anaplasma Capra*, an emerging tick-borne zoonotic pathogen. Vet Microbiol.

[CR67] Seo MG, Kwon OD, Kwak D (2020). Genotypic analysis of piroplasms and associated pathogens from ticks infesting cattle in Korea. Microorganisms.

[CR69] Shi K, Li J, Yan Y, Chen Q, Wang K, Zhou Y, Li D, Chen Y, Yu F, Peng Y, Zhang L, Ning C (2019). Dogs as new hosts for the emerging zoonotic pathogen *Anaplasma Capra* in China. Front Cell Infect Microbiol.

[CR70] Shi Y, Yang J, Guan G, Liu Z, Luo J, Song M (2020). Molecular investigation of *Anaplasma* species in sheep from Heilongjiang Province, northeast China identified four *Anaplasma* species and a novel genotype of *Anaplasma Capra*. Parasitol Int.

[CR71] Shin SU, Park YJ, Ryu JH, Jang DH, Hwang S, Cho HC, Park J, Han JI, Choi KS (2020). Identification of zoonotic tick-borne pathogens from Korean water deer (*Hydropotes inermis argyropus*). Vector Borne Zoonotic Dis.

[CR74] Staji H, Yousefi M, Hamedani MA, Tamai IA, Khaligh SG (2021). Genetic characterization and phylogenetic of *Anaplasma Capra* in Persian onagers (*Equus hemionus onager*). Vet Microbiol.

[CR72] Stuen S, Granquist EG, Silaghi C (2013). *Anaplasma phagocytophilum*—a widespread multi-host pathogen with highly adaptive strategies. Front Cell Infect Microbiol.

[CR73] Sun XF, Zhao L, Wen HL, Luo LM, Yu XJ (2015). *Anaplasma* species in China. Lancet Infect Dis.

[CR75] Teng Z, Shi Y, Zhao N, Zhang X, Jin X, He J, Xu B, Qin T (2023) Molecular detection of tick-borne bacterial and protozoan pathogens in *Haemaphysalis longicornis* (Acari: Ixodidae) ticks from free-ranging domestic sheep in Hebei Province, China. Pathogens 12(6): 763. 10.3390/pathogens12060763 10.3390/pathogens12060763 PMC10303831 37375453

[CR76] Teodorowski O, Radzki R, Kalinowski M, WIniarczyk S, Garcia Bocanegra I, Winiarczyk D, Adaszek L (2020). Molecular detection of *Anaplasma phagocytophilum* in roe deer (*Capreolus capreolus*) in eastern Poland. Ann Agric Environ Med.

[CR77] Theiler A (1910). *Anaplasma marginale* (Gen. and Spec. Nov.): the marginal points in the blood of cattle suffering from a specific disease.

[CR78] Torina A, Galindo RC, Vicente J, Di Marco V, Russo M, Aronica V, Fiasconaro M, Scimeca S, Alongi A, Caracappa S, Kocan KM, Gortazar C, de la Fuente J (2010). Characterization of *Anaplasma phagocytophilum* and *A. ovis* infection in a naturally infected sheep flock with poor health condition. Trop Anim Health Prod.

[CR79] Wang H, Yang J, Mukhtar MU, Liu Z, Zhang M, Wang X (2019). Molecular detection and identification of tick-borne bacteria and protozoans in goats and wild siberian roe deer (*Capreolus pygargus*) from Heilongjiang Province, northeastern China. Parasit Vector.

[CR80] Wang K, Yan Y, Zhou Y, Zhao S, Jian F, Wang R, Zhang L, Ning C (2021). Seasonal dynamics of *Anaplasma* spp. in goats in warm-temperate zone of China. Ticks Tick Borne Dis.

[CR81] Wang Y, Zhang Q, Han S, Li Y, Wang B, Yuan G, Zhang P, Yang Z, Zhang H, Sun Y, Chen J, Han X, He H (2021). *Ehrlichia chaffeensis* and four *Anaplasma* species with veterinary and public health significance identified in tibetan sheep (*Ovis aries*) and yaks (*Bos grunniens*) in Qinghai, China. Front Vet Sci.

[CR83] Wei W, Li J, Wang YW, Jiang BG, Liu HB, Wei R, Jiang RR, Cui XM, Li LF, Yuan TT, Wang Q, Zhao L, Xia LY, Jiang JF, Qiu YF, Jia N, Cao WC, Hu YL (2020). *Anaplasma platys*-like infection in goats, Beijing, China. Vector Borne Zoonotic Dis.

[CR82] Woldehiwet Z (2010) The natural history of *Anaplasma phagocytophilum*. Vet Parasitol 167(2–4). 10.1016/j.vetpar.2009.09.013. 108 – 22 10.1016/j.vetpar.2009.09.013 19811878

[CR84] Yan Y, Wang K, Cui Y, Zhou Y, Zhao S, Zhang Y, Jian F, Wang R, Zhang L, Ning C (2021) Molecular detection and phylogenetic analyses of *Anaplasma* spp. in *Haemaphysalis longicornis* from goats in four provinces of China. Sci Rep 11(1): 14155. 10.1038/s41598-021-93629-3 10.1038/s41598-021-93629-3 PMC8266805 34238975

[CR85] Yang J, Liu Z, Niu Q, Liu J, Han R, Liu G, Shi Y, Luo J, Yin H (2016). Molecular survey and characterization of a novel *Anaplasma* species closely related to *Anaplasma Capra* in ticks, northwestern China. Parasit Vector.

[CR86] Yang J, Liu Z, Niu Q, Liu J, Han R, Guan G, Hassan MA, Liu G, Luo J, Yin H (2017). A novel zoonotic *Anaplasma* species is prevalent in small ruminants: potential public health implications. Parasit Vector.

[CR87] Yang J, Liu Z, Niu Q, Mukhtar MU, Guan G, Liu G, Luo J, Yin H (2018). A novel genotype of *Anaplasma Capra* in wildlife and its phylogenetic relationship with the human genotypes. Emerg Microbes Infect.

[CR88] Zhang Y, Cui Y, Sun Y, Jing H, Ning C (2020). Novel *Anaplasma* variants in small ruminants from Central China. Front Vet Sci.

[CR89] Zhou M, Cao S, Sevinc F, Sevinc M, Ceylan O, Ekici S, Jirapattharasate C, Moumouni PF, Liu M, Wang G, Iguchi A, Vudriko P, Suzuki H, Xuan X (2017). Molecular detection and genetic characterization of *Babesia, Theileria* and *Anaplasma* amongst apparently healthy sheep and goats in the central region of Turkey. Ticks Tick Borne Dis.

[CR90] Zhou S, Huang L, Lin Y, Bhowmick B, Zhao J, Liao C, Guan Q, Wang J, Han Q (2023). Molecular surveillance and genetic diversity of *Anaplasma* spp. in cattle (*Bos taurus*) and goat (*Capra aegagrus hircus*) from Hainan island/province, China. BMC Vet Res.

